# Body Dissatisfaction and Disordered Eating Behaviors: The Mediation Role of Smartphone Addiction and Depression

**DOI:** 10.3390/nu14061281

**Published:** 2022-03-17

**Authors:** Fahui Yang, Le Qi, Shiyu Liu, Wen Hu, Qike Cao, Ying Liu, Man Tang, Zhuolan Lv, Zhehan Zhou, Yingkai Yang

**Affiliations:** 1 Faculty of Psychology, Southwest University, Chongqing 400715, China; fahui2019@swu.edu.cn (F.Y.); liushiyua@email.swu.edu.cn (S.L.); qike1225@email.swu.edu.cn (Q.C.); a985678@email.swu.edu.cn (Y.L.); tm20010730@email.swu.edu.cn (M.T.); wavelv@email.swu.edu.cn (Z.L.); zzh020521lg@email.swu.edu.cn (Z.Z.); 2 Department of Psychology, Chongqing City Management College, Chongqing 401331, China; zjs852314577@email.swu.edu.cn; 3 Mindrun Educational Technology Co., Ltd., Shenzhen 518000, China; whu@email.biai123.net

**Keywords:** disordered eating behaviors, body dissatisfaction, smartphone addiction, depression

## Abstract

This study aimed to determine whether smartphone addiction and depression sequentially mediate the relationship between body dissatisfaction and disordered eating behaviors (e.g., restrained eating, emotional eating and external eating). A total of 5986 participants (54.1% females, average age = 19.8 years, age range = 17–32) completed the Satisfaction and Dissatisfaction with Body Parts Scale, the Three-Factor Eating Questionnaire, the Smartphone Addiction Scale and the Patient Health Questionnaire-9. Mediational analysis showed that, after controlling for age, sex and body mass index, body dissatisfaction was related to disordered eating behaviors through (a) the mediating effect of smartphone addiction, (b) the mediating effect of depression, and (c) the serial mediating effect of smartphone addiction and depression. In conclusion, our study showed for the first time that smartphone addiction and depression can be sequential mediator variables in the association between body dissatisfaction and disordered eating. However, this study is a cross-sectional study; future longitudinal studies could further test the causal associations between these study variables.

## 1. Introduction

Disordered eating behaviors (e.g., restrained eating) are highly prevalent among adolescents and young adults [1]. In a recent survey comprising 14,322 Americans aged between 18 and 24, 19.1% of the participants were found to have engaged in dieting, with another 3.6% having used extreme weight loss methods [2]. A recent national survey reported that the age-standardized prevalence of screen-detected disordered eating in mainland China was estimated to be 7.04% in 2015 and has been increasing over the last decade [3]. More importantly, disordered eating behaviors—even those less severe or frequent than the disorders defined according to the Diagnostic and Statistical Manual of Mental Disorders (DSM-5) criteria [4]—are associated not only with increased risk of developing eating disorders, but also with other harmful consequences, such as insufficient nutritional intake and significant weight gain over time [1]. Therefore, it is critical to be aware of the factors that contribute to disordered eating behaviors.

Previous studies have identified body dissatisfaction as a risk factor for the development of disordered eating behaviors across different cultures [5,6,7]. Body dissatisfaction refers to the perceived discrepancy between one’s actual body image and one’s ideal body image [8]. Body dissatisfaction is pervasive among college students, since physical appearance—especially with regard to body shape—is a great concern of many at this particular stage of life [7,9]. Previous studies have found that individuals with higher levels of body dissatisfaction are more likely to engage in disordered eating behaviors such as dieting, unhealthy eating and weight control practices [10,11]. However, the exact pathway through which body dissatisfaction influences disordered eating behavior is yet to be clarified.

Body dissatisfaction has been found to be a proximal factor that directly contributes to disordered eating [5]. Specifically, disordered eating behaviors (e.g., strict dieting and restrained eating) may serve as a means of losing weight and eventually achieving the ideal body image. This is especially the case for female young adults, who have a tendency to diet in pursuit of a slimmer body shape due to sociocultural influences [7,12]. Meanwhile, recent reports have suggested that restrained eating is also prevalent among males due to a growing trend for muscle building [13,14].

On the other hand, there has been an accumulation of evidence to suggest that body dissatisfaction may influence disordered eating through certain mediating factors. Among others, negative affectivity (e.g., depression) is a potential candidate for mediating the effect of body dissatisfaction [15]. Negative affectivity has been repeatedly identified as a proximal factor implicated in adolescents’ various disordered eating behaviors [16,17]. For instance, it has been speculatively suggested that both restrained eating and binge eating are triggered by depressive feelings [15] and are a method of distracting oneself from negative self-perceptions or providing immediate emotional relief [18]. Furthermore, the degree of body dissatisfaction in different age groups has been documented as being strongly associated with the severity of depressive symptoms [17,19,20]. For adolescents and young adults, bodily appearance is central to one’s self-evaluation [21], and individuals with high levels of body dissatisfaction may have negative feelings about their own appearance and, accordingly, may anticipate possible negative evaluations from others in social settings [22]. As proposed by the cognitive theories of depression, this negative self-evaluation is central to the development of depression [23]. Accordingly, it is reasonable to hypothesize that depression could mediate the effect of body dissatisfaction on disordered eating behaviors.

Smartphone addiction has also emerged as a potential mediating factor. Recent developments in mobile devices have enabled users to access the internet for a wide range of purposes, at a time and location of one’s own choosing [24,25]. As a result, users have become increasingly preoccupied with their smartphones, which is highly likely to have an impact on their daily lives and health [26]. Recent studies have begun to reveal smartphone addiction as being a predictor of various eating disorders [25,27,28,29]. Unhealthy or excessive mobile phone use may encourage a sedentary lifestyle which in turn may lead to an insufficient intake of healthy food and a high intake of junk food or fast food [30]. In addition, the frequent use of social networks via mobile devices may promote the internalization of certain ideals pertaining to body image, and may motivate an individual to take action in order to achieve these ideals [31]. Unhealthy eating practices—such as restricting food intake—could thus be exacerbated and eventually lead to disordered eating behaviors. Supporting this idea are the findings of Tayhan Kartal and Yabanci Ayhan (2021) [25], who showed that the Smartphone Addiction Test score is positively associated with the Eating Attitude Test-40 (*r* = 0.277). Furthermore, cellphone addiction has been shown to be a risk factor for various psychological disorders, including depression. For example, A recent meta-analysis showed that problematic smartphone usage was associated with increased likelihood of depression (odds ratio = 3.17), anxiety (odds ratio = 3.05) and higher perceived stress (odds ratio = 1.86) [32]. This further supports the previously stated notion that the association between smartphone addiction and depression may in turn lead to disordered eating behaviors.

Finally, body dissatisfaction has been suggested to have a positive correlation with smartphone addiction among adolescents [8]. According to the cognitive-behavioral model of internet addiction [33], individuals with negative self-evaluation are more vulnerable to addiction in that these individuals aim to elicit positive responses by selectively focusing on and presenting the positive aspects of themselves and/or by seeking reassurance from others in social interactions. As mentioned above, body appearance is of great concern to adolescents and, accordingly, body image is central to their self-evaluation [21]. Similarly, the compensatory satisfaction theory [34] proposes that individuals with high levels of body dissatisfaction tend to use smartphones as a compensatory approach to satisfy their psychological demands, which are difficult to perceive in reality. Consequently, problematic mobile phone use may be repeatedly reinforced by a feeling of satisfaction.

To summarize, it was the aim of this study to explore the relationship in Chinese young adults between body dissatisfaction and three types of disordered eating behaviors, namely, cognitive restraint eating (the tendency for individuals to monitor and restrict their eating in order to lose weight), emotional eating (the tendency to eat in response to negative emotions) and external eating (the tendency to overeat with a sense of being out of control). We hypothesized that body dissatisfaction was positively associated with the disordered eating behaviors listed above, with similar patterns emerging in terms of (a) the mediating effect of depression, (b) the mediating effect of smartphone addiction and (c) the serial mediating effect of smartphone addiction and depression.

## 2. Method

### 2.1. Participants

College students were recruited from four provinces of China, including Chongqing, Zhejiang, Guangdong and Shandong. The counselors (who oversee the learning and life of students) sent the link for our questionnaire to their students. In total, 5986 participants (54.1% females) voluntarily took part and completed the anonymous online survey. Their mean age was 19.8 (*SD* = 1.75), with an age range of 17 to 32. Their mean body mass index (BMI) was 20.40 (*SD* = 2.97), with a BMI range of 14.42 to 37.34. A total of 76.7% (*n* = 4591) of the participants were underweight or of normal weight (e.g., BMI below 24 kg/m^2^), 6.8% (*n* = 413) were overweight (BMI between 24 and 27.99 kg/m^2^) and 2.3% (*n* = 140) were obese (BMI equal to or above 30 kg/m^2^). However, 842 participants did not report their height or weight, and therefore their BMI information was missing.

### 2.2. Measures

#### 2.2.1. Body Dissatisfaction

Body dissatisfaction was measured using the Satisfaction and Dissatisfaction with Body Parts Scale [35], which asks participants to indicate their levels of satisfaction for 9 body parts (e.g., waist, thighs). The 9 items were scored on a 5-point Likert scale ranging from 1 (extremely satisfied) to 5 (extremely dissatisfied). All items were summed to create an index, with higher scores indicating higher levels of body dissatisfaction. The Cronbach coefficient of this scale in the study was 0.95.

#### 2.2.2. Smartphone Addiction

Smartphone addiction was measured using the Smartphone Addiction Scale–Short Version [26]. This 10-item scale assesses smartphone addiction using a 6-point Likert scale ranging from 1 (strongly disagree) to 6 (strongly agree). All items were summed to create an index, with higher scores indicating higher risks of smartphone addiction. The Cronbach coefficient of this scale in the study was 0.95.

#### 2.2.3. Depression

Depression was measured using the Patient Health Questionnaire [36]. This 9-item scale asks participants to rate how they have been feeling over the previous 2 weeks. Each question is scored from 0 to 3 (0 = not at all, 1 = several days, 2 = more than half the days and 3 = nearly every day), with higher scores indicating higher levels of depression. The Cronbach coefficient of this scale in the study was 0.91.

#### 2.2.4. Disordered Eating Behaviors

Disordered eating behaviors were measured using the Dutch Eating Behavior Questionnaire [37]. This 33-item scale measures emotional (13 items), external and restrained eating (10 items each). Each question is scored from 1 (never) to 5 (very often) with higher scores indicating higher levels of disordered eating behaviors. Examples of items were as follows: “Do you have a desire to eat when you are emotionally upset?” (emotional eating); “If food tastes good to you, do you eat more than usual?” (external eating); and “How often do you try not to eat in the evening because you are watching your weight?” (restrained eating). The Cronbach coefficient of the subscales in the study were 0.95 (restrained eating), 0.97 (emotional eating) and 0.90 (external eating).

#### 2.2.5. Covariates

Considering that age, sex and BMI are all important factors associated with disordered eating behaviors, these variables were treated as covariates. Age, sex, weight and height were self-reported. The BMI was calculated using the standard formula of weight (kilograms) divided by height (meters) squared (BMI = kg/m^2^).

### 2.3. Statistical Analysis

In this study, descriptive statistics, Harman’s single factor test and Pearson’s correlation/point-biserial correlation analyses were completed using SPSS 26.0 (Armonk, New York, NY, USA). Mediation analyses were performed to determine the indirect role of smartphone addiction and depression in the relationship between body dissatisfaction and disordered eating behaviors (e.g., restrained eating, external eating and emotional eating). The mediation analyses were performed using R version 3.6.2 (Vienna, Austria) and the lavaan R package version 0.6-9 (Vienna, Austria). All mediation analyses were adjusted for covariates. Because there were missing values for BMI, full information maximum likelihood was used to handle them. The significance of the mediation effects was analyzed using the bootstrap resampling method. The number of bootstraps was set at 5000. A significant mediation occurred when the 95% confidence interval for the index of mediation did not contain zero.

## 3. Results

### 3.1. Common Method Bias Analysis

Because all of the data in our study were gathered from self-reported questionnaires, we conducted Harman’s single factor test to examine the common method bias. The results showed that the first principal factor explained 33.69% of the variance (e.g., below 50%), indicating that there were no significant issues with our present study concerning common method biases for estimates of the associations among the study variables [38].

### 3.2. Preliminary Analyses

The descriptive statistics and the correlation matrix for the study variables are presented in Table 1.

### 3.3. Testing for Mediation Model

**Figure 1:**
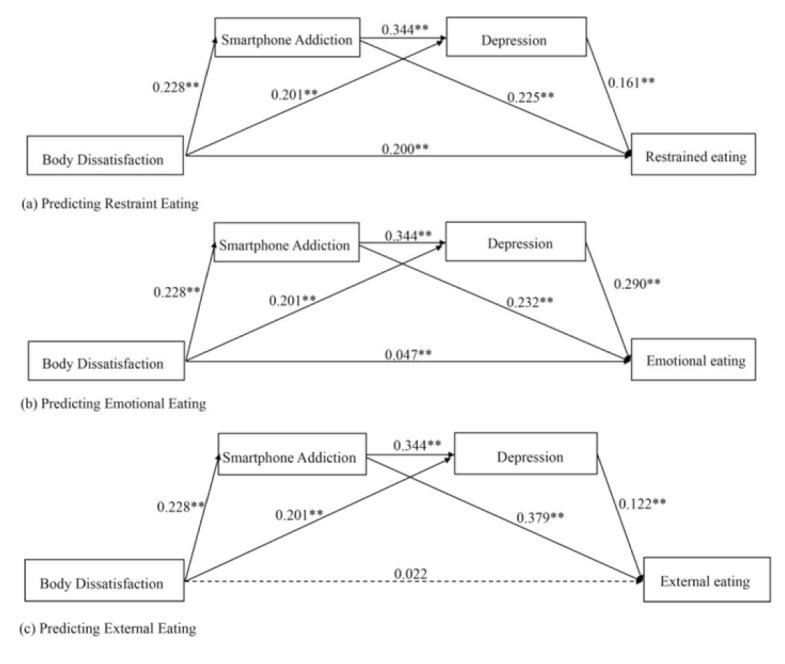
Multiple mediation models predicting (**a**) restrained eating, (**b**) emotional eating and (**c**) external eating from body dissatisfaction, smartphone addiction and depression. All path coefficients are standardized. ** *p* < 0.01.

Table 2 and Figure 1 show the mediation results (see Supplementary Material Tables S1 and S2 for the separate mediation results for the male and female samples). Smartphone addiction was found to mediate the relationships between body dissatisfaction and restrained eating (indirect effect = 0.05, 95% CI = 0.04–0.06, *p* < 0.001), emotional eating (indirect effect = 0.05, 95% CI = 0.05–0.06, *p* < 0.001) and external eating (indirect effect = 0.09, 95% CI = 0.08–0.10, *p* < 0.001). The indirect effects of smartphone addiction accounted for 17.4% (restrained eating, proportion mediated = 0.17, 95% CI (0.15, 0.20), *p* < 0.001), 60.6% (emotional eating, proportion mediated = 0.61, 95% CI (0.50, 0.72), *p* < 0.001) and 29.2% (external eating, proportion mediated = 0.29, 95% CI (0.24, 0.35), *p* < 0.001) of the variances explained in disordered eating behaviors by body dissatisfaction.

Similarly, depression mediated the relationships between body dissatisfaction and restrained eating (indirect effect = 0.03, 95% CI = 0.03–0.04, *p* < 0.001), emotional eating (indirect effect = 0.06, 95% CI = 0.05–0.07, *p* < 0.001) and external eating (indirect effect = 0.02, 95% CI = 0.02–0.03, *p* < 0.001). The indirect effects of depression accounted for 10.9% (restrained eating, proportion mediated = 0.11, 95% CI (0.09, 0.13), *p* < 0.001), 17.2% (emotional eating, proportion mediated = 0.17, 95% CI (0.12, 0.22), *p* < 0.001) and 32.3% (external eating, proportion mediated = 0.32, 95% CI (0.26, 0.38), *p* < 0.001) of the variances explained in disordered eating behaviors by body dissatisfaction.

Moreover, mobile phone addiction and depression sequentially mediated the links between body dissatisfaction and restrained eating (sequential indirect effect = 0.01, 95% CI = 0.01–0.02, *p* < 0.001), emotional eating (sequential indirect effect = 0.02, 95% CI = 0.02–0.03, *p* < 0.001), and external eating (sequential indirect effect = 0.01, 95% CI = 0.007–0.012, *p* < 0.001). The sequential indirect effects accounted for 13.1% (restrained eating, proportion mediated = 0.13, 95% CI (0.11, 0.15), *p* < 0.001), 7.9% (emotional eating, proportion mediated = 0.08, 95% CI (0.07, 0.09), *p* < 0.001), and 17.0 % (external eating, proportion mediated = 0.17, 95% CI (0.15, 0.19), *p* < 0.001) of the total indirect effects.

In short, our results show that smartphone addiction and depression play (sequential) mediating roles in the associations between body dissatisfaction and disordered eating behaviors.

## 4. Discussion

The current study examines the association between body dissatisfaction and disordered eating behaviors among Chinese college students. The mediating effects of smartphone addiction and depression on this association were also investigated. Our data demonstrates that body dissatisfaction is positively associated with the disordered eating behaviors of restrained eating, emotional eating and external eating. Consistent with previous studies [6,15], our results confirmed this association to be mediated by depression. Additionally, we were able to show for the first time that body dissatisfaction could impact the three disordered eating behaviors through (a) the mediating effect of smartphone addiction and (b) the serial mediating effect of smartphone addiction and depression.

The mediating role of depression in the relationship between body dissatisfaction and disordered eating behaviors has been previously documented. For example, Cruz-Saez et al. (2020) [6] reported that body dissatisfaction among adolescents was positively correlated with their EDI-2 (Eating Disorder Inventory-2) score and this effect was directly mediated by negative affectivity, including depression. Similarly, Brechan and Kvalem (2015) [15] found that depression was a significant mediator in the relationship of body dissatisfaction with both binge eating and restrained eating in women. Following on from these previous investigations, our study illustrates that depression mediates the influence of body dissatisfaction on restrained eating, emotional eating and external eating in Chinese college students. Specifically, college students with high levels of body dissatisfaction are more likely to experience depression, perhaps due to the considerable discrepancies between perceived body shapes and ideal body shapes that are usually too unrealistic to easily achieve [39]. Depression could, in turn, result in an increased likelihood of disordered eating behaviors, including restrained eating, emotional eating and external eating. According to the transdiagnostic model of eating disorders, disordered eating behaviors can be understood as maladaptive responses for coping with or distracting oneself from distressing emotions [40]. Therefore, it is important to note that the prevention and treatment of eating disorders as well as disordered eating behaviors should incorporate strategies that directly target the regulation of negative emotions.

This study also examined the potential mediating role of smartphone addiction in the relationship between body dissatisfaction and disordered eating. Due to the high prevalence of smartphone use, the etiology and consequences of smartphone addiction have attracted considerable academic interest in recent years. Previous studies have documented the individual associations of smartphone addiction with disordered eating behaviors [25,27,28,41], distressing emotions [42,43] and body dissatisfaction [8,44]. For example, Liu et al. (2020) [8] recently reported that body dissatisfaction could positively predict adolescent smartphone addiction. Problematic smartphone usage was associated with an increased likelihood of depression, anxiety and higher levels of perceived stress, according to a recent meta-analysis study [32]. A longitudinal study covering the period from adolescence to emerging adulthood showed that early problematic cell phone use predicted depression later on [42]. Tayhan Kartal and Yabanci Ayhan (2021) [25] showed that smartphone addiction was positively associated with the Eating Attitude Test-40 scores of college students.

Building upon these prior studies, this study further establishes smartphone addiction as a novel mediator in the association between body dissatisfaction and the three disordered eating behaviors. According to the compensatory satisfaction theory, the problematic use of smartphones could be considered a compensatory strategy to satisfy psychological demands that cannot be met in reality [34,45], because in the virtual world, an individual may temporarily be able to mitigate dissatisfaction by selectively presenting the positive aspects of themselves [46] and constantly seeking reassurance from others [47]. Thus, habitual behaviors are reinforced and, in the long term, the risk of other detrimental behaviors such as disordered eating may be increased.

Smartphone addiction could be directly related to disordered eating behaviors. There are important neurocognitive similarities between addictive behavior (e.g., smartphone addiction) and eating dysregulation (e.g., restrained eating and external eating) [48,49,50,51,52]. For example, both smartphone addiction and disordered eating behaviors are related to higher reward sensitivity [51,53] and impulsivity [54,55]. These shared mechanisms could explain the higher correlations observed between smartphone addiction and the disordered eating behaviors in this study.

In addition, these significant associations between smartphone addiction and disordered eating behaviors could also be a result of an increase in lifestyles based on the adoption of new technologies. Excessive smartphone use may, for example, bring about a reduction in physical activities and encourage a more sedentary lifestyle, which may, in turn, lead to unhealthy eating habits such as skipping meals, excessive fast food consumption and insufficient intake of healthy foods [25,27]. To support these inferences, a systematic review has shown that there is a negative relationship between excessive smartphone use and physical activity [56]. Importantly, individuals with disordered eating behaviors exhibited more sedentary behavior and less physical activity [57].

Our data indicate that smartphone addiction could also indirectly influence eating behaviors through depression. We speculate that frequent use of the internet via smartphones may reinforce the internalization of ideal body shapes. However, mainstream ideals are usually very difficult to achieve; thus, depression may be induced in students through social comparison [5]. Finally, as mentioned above, young adults tend to engage in disordered eating behaviors in order to cope with depressive emotions [16].

To summarize, our study has extended the scope of previous research by revealing the novel mediating role of smartphone addiction as well as the serial mediating role of smartphone addiction and depression in the relationship between body dissatisfaction and disordered eating behaviors in Chinese college students. These findings have important clinical implications for the prevention and treatment of restrained eating, emotional eating and external eating, as well as other disordered eating behaviors. It could be proposed that smartphone addiction be included in the conceptualization of disordered eating. Accordingly, strategies that target the management of smartphone usage should be considered and implemented to provide a comprehensive intervention. In addition, interventions might also benefit from a particular focus on the cognitive factors (e.g., body judgments and the internalization of unrealistic physical standards) induced by social media and smartphone use, given that these factors have been shown to mediate the relationship between smartphone use and body-related or eating-related outcomes [58,59].

## 5. Limitations and Future Research Directions

Notably, this study has several limitations. Firstly, it is a cross-sectional study; consequently, it does not consider the causal relationships between the variables investigated (body dissatisfaction, smartphone addiction, depression and the three disordered eating behaviors). Future longitudinal studies are necessary to shed light on the causal and temporal relationships among the variables. Secondly, despite the large sample size, our study focused solely on college students, thus representing only a specific portion of the Chinese young adult population. Therefore, the generalizability of these results is limited. It is necessary for future studies to recruit a more diverse range of participants, from various educational and cultural backgrounds. Meanwhile, further investigations among other age groups—such as adolescents—would be highly valuable. Thirdly, all of the data were collected through the medium of self-report questionnaires; therefore, the potential influence of subjectivity in the participants’ responses cannot be ruled out. Future studies could benefit from combining self-report measures with more objective methods for quantifying individuals’ smartphone use (e.g., monitoring software) in an effort to limit common-method variance. Finally, we omitted to assess other important variables related to body dissatisfaction or disordered eating behaviors, such as sexual orientation. More studies are needed to examine the theoretical models of disordered eating behavior that incorporate personological aspects, mood dimension and lifestyles.

## 6. Conclusions

In conclusion, our research demonstrates that a consideration of the roles of smartphone addiction and depression is crucial in order to more fully understand the association between body dissatisfaction and disordered eating behaviors among Chinese college students. The findings indicate that interventions that target smartphone addiction and the various emotions relating to depression could be of great value for mitigating the detrimental effects of body dissatisfaction on disordered eating behaviors.

## Figures and Tables

**Table 1:** Correlations, means and standard deviations of study variables.

Variables	*M* (*SD*)	Correlations								
		1	2	3	4	5	6	7	8	9
1. Age	19.83 (1.75)	—								
2. Sex	—	−0.02	—							
3. BMI	20.40 (2.97)	0.00	0.33 **	—						
4. Body dissatisfaction	43.69 (8.38)	−0.04 **	−0.21 **	0.20 **	—					
5. Smartphone addiction	28.2 (11.64)	−0.05 **	−0.18 **	−0.03	0.25 **	—				
6. Depression	12.97 (4.63)	−0.01	−0.10 **	−0.02	0.27 **	0.39 **	—			
7. Restrained eating	17.63 (8.33)	−0.05 **	−0.23 **	0.20 **	0.39 **	0.37 **	0.32 **	—		
8. Emotional eating	21.8 (10.88)	−0.04 **	−0.24 **	0.00	0.23 **	0.39 **	0.41 **	0.49 **	—	
9. External eating	25.24 (8.71)	−0.03 **	−0.07 **	−0.07 **	0.20 **	0.47 **	0.30 **	0.42 **	0.61 **	—

Note: BMI = body mass index; *M* = mean, *SD* = standard deviation. Sex was dummy coded such that male = 1 and female = 0. ** *p* < 0.01. The correlations of sex with other continuous variables are point-biserial correlations.

**Table 2:** Summary of indirect effects from body dissatisfaction to disordered eating behaviors.

	Coefficient	SE	95% CI	*p*
Indirect effects (via mediators)				
BD→MP→RE	0.051	0.004	0.043, 0.059	<0.001
BD→DP→RE	0.032	0.003	0.026, 0.039	<0.001
BD→MP→DP→RE	0.013	0.001	0.010, 0.015	<0.001
BD→MP→EE	0.053	0.004	0.045, 0.061	<0.001
BD→DP→EE	0.058	0.004	0.049, 0.067	<0.001
BD→MP→DP→EE	0.023	0.002	0.019, 0.026	<0.001
BD→MP→External E	0.086	0.004	0.075, 0.098	<0.001
BD→DP→External E	0.024	0.004	0.019, 0.030	<0.001
BD→MP→DP→External E	0.010	0.002	0.007, 0.012	<0.001

Note: SE = standard error; BD = body dissatisfaction; MP = smartphone addiction; DP = depression; RE = restrained eating; EE = emotional eating; External E = external eating.

## Data Availability

Datasets arising from the study might be available upon reasonable request from the corresponding author.

## Supplementary Materials

The following supporting information can be downloaded at: https://www.mdpi.com/article/10.3390/nu14061281/s1, Table S1: Summary of indirect effects from body dissatisfaction to disordered eating behaviors in male sample.; Table S2: Summary of indirect effects from body dissatisfaction to disordered eating behaviors in female sample.
