# Supplementary material for: Body Dissatisfaction and Disordered Eating Behaviors: The Mediation Role of Smartphone Addiction and Depression
Source: Nutrients. 2022 Mar 17;14(6):1281. doi: 10.3390/nu14061281 (PMC8955505; doi:10.3390/nu14061281)
Supplement: Supplementary file 1 [file nutrients-14-01281-s001.zip › nutrients-1602786-supplementary-done.pdf]

nutrients MDPI

Supplementary material for

Body Dissatisfaction and Disordered Eating Behaviors: The Mediation Role of Smartphone Addiction and Depression

Table S1. Summary of indirect effects from body dissatisfaction to disordered eating behaviors in male sample.

	Coefficient	S.E	95% CI	*P*
Indirect effects (via mediators)				
BD→MP→RE	0.041	0.005	0.031, 0.051	<0.001
BD→DP→RE	0.033	0.004	0.025, 0.042	<0.001
BD→MP→DP→RE	0.011	0.002	0.010, 0.015	<0.001
BD→MP→EE	0.069	0.008	0.054, 0.061	<0.001
BD→DP→EE	0.025	0.004	0.017, 0.085	<0.001
BD→MP→DP→EE	0.008	0.001	0.006, 0.033	<0.001
BD→MP→External E	0.040	0.005	0.030, 0.050	<0.001
BD→DP→External E	0.054	0.006	0.043, 0.065	<0.001
BD→MP→DP→External E	0.018	0.002	0.014, 0.023	<0.001

Note：S.E= standard error; BD=body dissatisfaction; MP= smartphone addiction; DP =depression; RE= restraint eating; EE= emotional eating; External E = external eating.

Table S2. Summary of indirect effects from body dissatisfaction to disordered eating behaviors in female sample.

	Coefficient	S.E	95% CI	*P*
Indirect effects (via mediators)				
BD→MP→RE	0.063	0.007	0.050, 0.077	<0.001
BD→DP→RE	0.031	0.005	0.022, 0.040	<0.001
BD→MP→DP→RE	0.014	0.002	0.010, 0.018	<0.001
BD→MP→EE	0.069	0.007	0.055, 0.083	<0.001
BD→DP→EE	0.063	0.007	0.050, 0.077	<0.001
BD→MP→DP→EE	0.028	0.003	0.023, 0.034	<0.001
BD→MP→External E	0.107	0.009	0.090, 0.124	<0.001
BD→DP→External E	0.024	0.004	0.016, 0.032	<0.001
BD→MP→DP→External E	0.011	0.002	0.007, 0.014	<0.001

Note：S.E= standard error; BD=body dissatisfaction; MP= smartphone addiction; DP =depression; RE= restraint eating; EE= emotional eating; External E = external eating.
